# Assessment of aortic and iliac artery calcification using CT-angiography in kidney transplant candidates

**DOI:** 10.1186/s42155-025-00542-1

**Published:** 2025-05-06

**Authors:** Ola Sobhy A. Elmeseiny, Simon Winther, Hanne Skou Jørgensen, My Svensson, Morten Bøttcher, Per Ivarsen, Gratien Andersen, Henrik Birn, Marie Bodilsen Nielsen

**Affiliations:** 1 Department of Biomedicine, Aarhus University, Aarhus, Denmark; 2 Department of Renal Medicine, Aarhus University Hospital, Aarhus, Denmark; 3 Department of Cardiology, Gødstrup Hospital, Herning, Denmark; 4 Department of Nephrology, Aalborg University Hospital, Aalborg, Denmark; 5 Department of Clinical Medicine, Aarhus University, Aarhus, Denmark; 6 Department of Radiology, Aarhus University Hospital, Aarhus, Denmark

## Abstract

**Purpose:**

Assessment of vascular calcification provides the opportunity for risk stratification in kidney transplant candidates (KTCs), as vascular calcification constitutes an independent risk factor for cardiovascular events. The aim of the present study is to explore the feasibility of contrast enhanced computed tomography (CT)-angiography to quantitate vascular calcification, to avoid the extra radiation of an additional non-contrast CT scan.

**Methods and materials:**

43 KTCs who underwent concomitant non-contrast CT scans and CT-angiographies of the infrarenal aorta and iliac arteries were included. Vascular calcification was quantified using the Agatston method on non-contrast CT and applying individual Hounsfield Unit thresholds on CT-angiographies based on the radio density of the aortic lumen. The calcium scores and volumes from non-contrast CT scans and CT-angiographies were compared using linear regression and Bland–Altman plots.

**Results:**

Non-contrast CT revealed vascular calcification in the infrarenal aorta in 92% of KTCs and in the iliac arteries in 90% of KTCs. The calcium scores estimated from CT-angiography correlated linearly with the calcium scores based on non-contrast CT scans (infrarenal aorta: R^2^ = 0.71, p < 0.0001; iliac arteries: R^2^ = 0.71, p < 0.0001); however, the calcium scores were higher, and volumes were lower compared to the non-contrast CT scans. The median differences in calcium scores were 1517 [48 – 6138] for the infrarenal aorta, and 2361 [59 – 8644] for the iliac arteries.

**Conclusion:**

Vascular calcification is present in the majority of KTCs. Calcification of the infrarenal aorta and iliac arteries may be assessed using CT-angiography, though higher calcium scores and lower volumes are found compared to the non-contrast CT scan.

**Supplementary Information:**

The online version contains supplementary material available at 10.1186/s42155-025-00542-1.

## Introduction

Cardiovascular disease is a common cause of morbidity and mortality in patients with chronic kidney disease (CKD) [1–4]. Notably, in the context of kidney transplantation, cardiovascular events are the most frequent cause of post-transplant mortality and graft loss [5]. This highlights the importance of cardiovascular risk assessment and possible preventive measures in kidney transplant candidates.

Vascular calcifications are highly prevalent in kidney transplant candidates [6] and constitute an independent risk factor for cardiovascular events in this high-risk group [7–11]. Coronary artery calcium quantification performed on non-contrast computed tomography (CT) scans provides prognostic information on the risk of major adverse cardiovascular events in patients with CKD [3, 12, 13]. Previous studies have suggested a similar prognostic value of assessing calcification in the abdominal aorta [10, 14–17].

While non-contrast CT scans allow for assessment of vascular calcification [18, 19], contrast enhanced CT-angiography has become an essential tool for operative planning for vascular surgery allowing for evaluation of vessel stenosis [20, 21]. However, assessment of vascular calcification from CT-angiography is not validated. Nevertheless, recent studies have pointed to the feasibility of coronary artery calcification assessment from CT-angiography [22–24]. Coronary artery calcium scoring from non-contrast CT scans is based on an established methodology [25], whereas quantification of calcification in other vascular beds is not yet part of routine assessment.

The atherosclerotic burden in the iliac arteries is especially important in patients undergoing evaluation for kidney transplantation, as the anatomical conditions of the iliac arteries are essential for the arterial anastomosis of the transplanted kidney graft. Extensive calcification of the iliac arteries is a major risk factor for post-transplant complications [26–30]. It may contraindicate a transplantation as the location and severity of the calcification may make the graft implantation impossible [31]. Therefore, testing for vascular disease is recommended in kidney transplant candidates [32–34] and may include imaging; however, the most optimal modality has not been identified [35]. Expanding the utility of contrast enhanced CT-angiographies to also include calcium score estimation, can minimize the need for an additional non-contrast CT scan, thus diminishing radiation exposure.

Calcium scores and volumes from concomitantly performed non-contrast CT scans and CT-angiographies were compared, with the aim to explore the comparability of contrast enhanced CT-angiography to non-contrast CT scans for quantification of vascular calcification in the aorta and iliac arteries in kidney transplant candidates.

## Materials and methods

### Study population

**Fig. 1:**
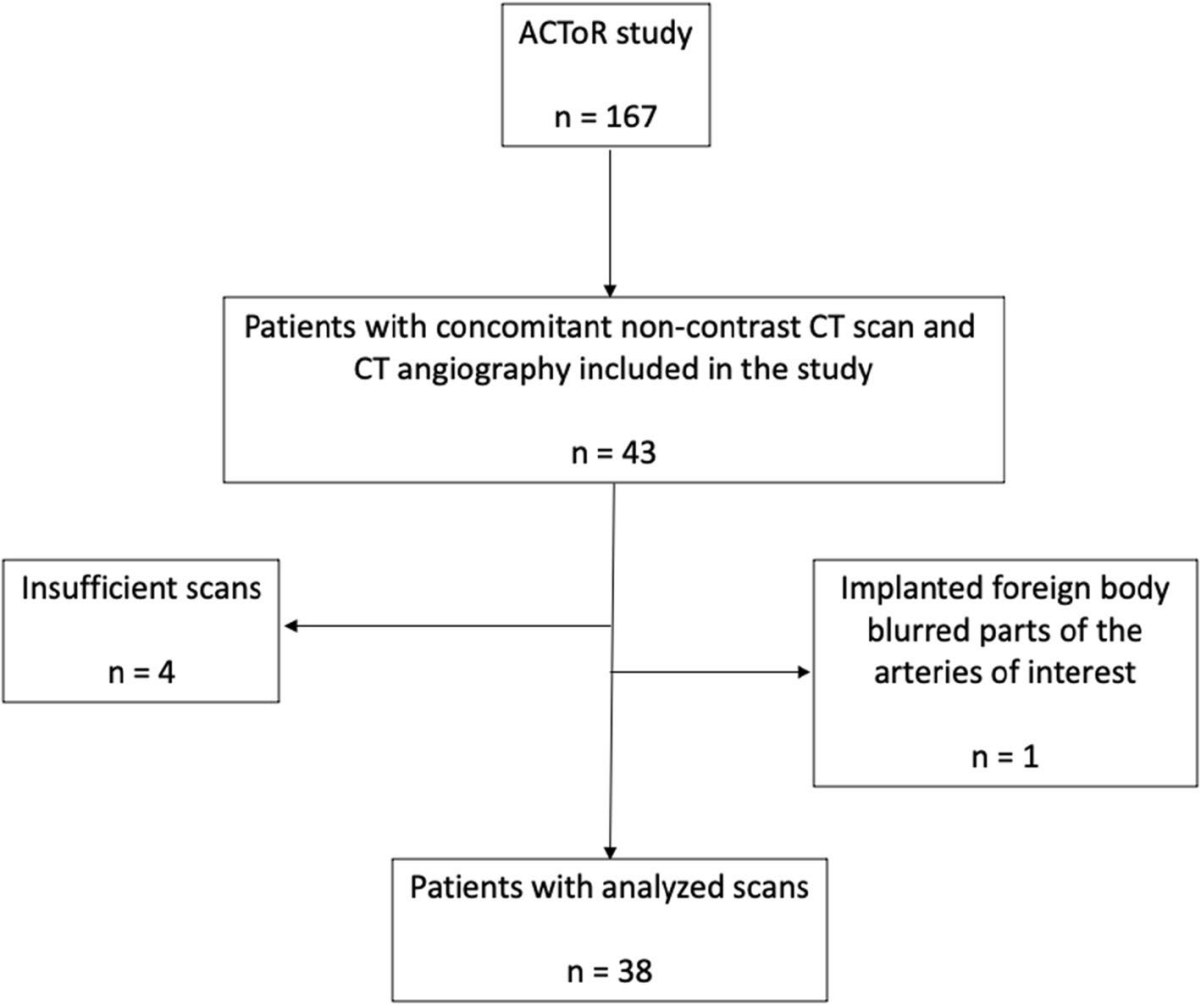
Flowchart of patient inclusion

The study population included patients from the prospective ACToR study cohort [36, 37] who underwent same day non-contrast CT scan and CT-angiography of both the infrarenal aorta and iliac arteries (Fig. 1). The ACToR cohort comprised kidney transplant candidates systematically referred for cardiovascular screening prior to kidney transplantation. Inclusion criteria were: age > 40 years, diabetes mellitus, dialysis treatment for > 5 years, or registered on kidney transplantation waiting list for > 3 years without cardiac screening [37]. Baseline characteristics were obtained from medical records and by patient interviews before the scan. The study was approved by the Central Denmark Region Ethics [1-45-70-33-20].

### Image acquisition

Patients underwent a CT scan of the abdomen and pelvis using a dual-source scanner (SOMATOM Definition Flash; Siemens Healthcare, Erlangen, Germany) including both a non-contrast and a contrast media-enhanced scan [36] (Table 1). The pitch was 3.4. For the CT-angiography, intravenous contrast doses were fixed at 95 mL for all patients (ioversol, 350 mg/mL; Mallinckrodt, Chesterfield, UK). The delay between contrast administration and image acquisition was 30 ± 5 s. The tube X-ray energy was set at 100 or 120 kVp depending on patient size. The median radiation dose was 1.3 [1.1–1.7] mSv for non-contrast CT scan and 3.6 [2.9–4.5] mSv for CT-angiographies, respectively.

**Table 1:** Parameters for performing and analyzing non-contrast CT scans and CT-angiographies

Variable	Non-contrast CT scans	CT-angiography
Slice thickness (mm)	3	3
Pitch	3.4	3.4
Contrast	None	95 mL ioversol
Kilovoltage (kVp)^a^	100 or 120	100 or 120
mSv	1.3 [1.1–1.7]	3.6 [2.9–4.5]
Minimum calcification area (mm^2^)	≥ 1	≥ 2
Attenuation threshold (HU)	Fixed 130 HU	Individual thresholds Mean HU + 3 SD

^a^Depending on patient size. HU = Hounsfield unit. SD = standard deviation

### Image analyses

For each patient, both the CT-angiography and the non-contrast CT scan were analyzed. The two scans per patient were evenly divided between two reviewers, so that each reviewer analyzed only one scan per patient. The reviewers were blinded to results of the paired scan, as well as the patient medical history.

Vascular calcification was quantified in the infrarenal aorta and the iliac arteries (common iliac arteries and external iliac arteries). The infrarenal aorta was defined as starting below the superior mesenteric artery branching and terminating at the level of the bifurcation into the common iliac arteries, both evaluated visually.

The interobserver variation was evaluated by randomly selecting 10 non-contrast CT scans and 10 CT-angiographies for re-analysis by the other reviewer. A time interval of several months was ensured between the original analysis of the paired scans and the re-analysis of the given scans.

The scan and analysis parameters are shown in Table 1. The scans were analyzed using Phillips IntelliSpace (Philips, Amsterdam, the Netherlands). Each of the non-overlapping slices were visually evaluated by the reviewers, and any calcium deposit was manually delineated in each slice to avoid including the spinal column as part of the calcification. The cumulated calcium values as calculated by the software were noted separately for the infrarenal aorta and for the iliac arteries. The calcium quantification was calculated as a calcium score and a calcium volume. The calcium score was measured using the Agatston method. For the non-contrast CT scans, a standard attenuation threshold of 130 Hounsfield Units (HU) was applied when measuring the Agatston score [25]. The Agatston score was automatically calculated by the software by multiplying the density score and the given area. The density score is defined depending on the HU: HU 130–199 = 1; HU 200–299 = 2; HU 300–399 = 3; and HU ≥ 400 = 4. The total score is a summation of all the given areas.

When analyzing the CT-angiographies, an individual attenuation threshold for each patient was applied based on the amount of contrast in the arterial lumen. The individual threshold was defined as the mean HU + 3 standard deviations (SD) of the contrast-enhanced aorta lumen. The mean HU of the aorta lumen was defined by delineating the lumen by a centrally placed circle with an area of 5 mm^2^ in the aorta lumen below the branching of the superior mesenteric artery. This threshold appeared to meet the criteria of including all the calcium and excluding the contrast, when different thresholds were evaluated. The calcium score of the CT-angiography was automatically calculated by the software using the same algorithm as for non-contrast CT scans, but with the individual thresholds (e.g. individual threshold of 250: HU 250–299 = 2; HU 300–399 = 3; and HU ≥ 400 = 4).

### Statistical analysis

Continuous variables are expressed as mean ± SD or median [interquartile range (IQR)] depending on distribution. Categorical variables are expressed as frequencies (%). The calcium scores and volumes were transformed to obtain normal distribution using the common logarithm (log 10(value + 1)). The coefficient of variance (CV%) and the interobserver variability (Pearson’s correlation) were calculated for the calcium scores obtained from non-contrast CT scans (n = 10) and CT-angiographies (n = 10), respectively. The association between calcium values (scores and volumes) obtained from the non-contrast CT scans and the CT-angiographies were evaluated by linear regression. The agreement between the methods was analyzed using XY and Bland–Altman plots. Statistical analyses were performed using Microsoft® Excel (©Microsoft, Redmond, USA) and Stata® Version 14.2 (StataCorp, College Station, Texas).

## Results

**Fig. 2:**
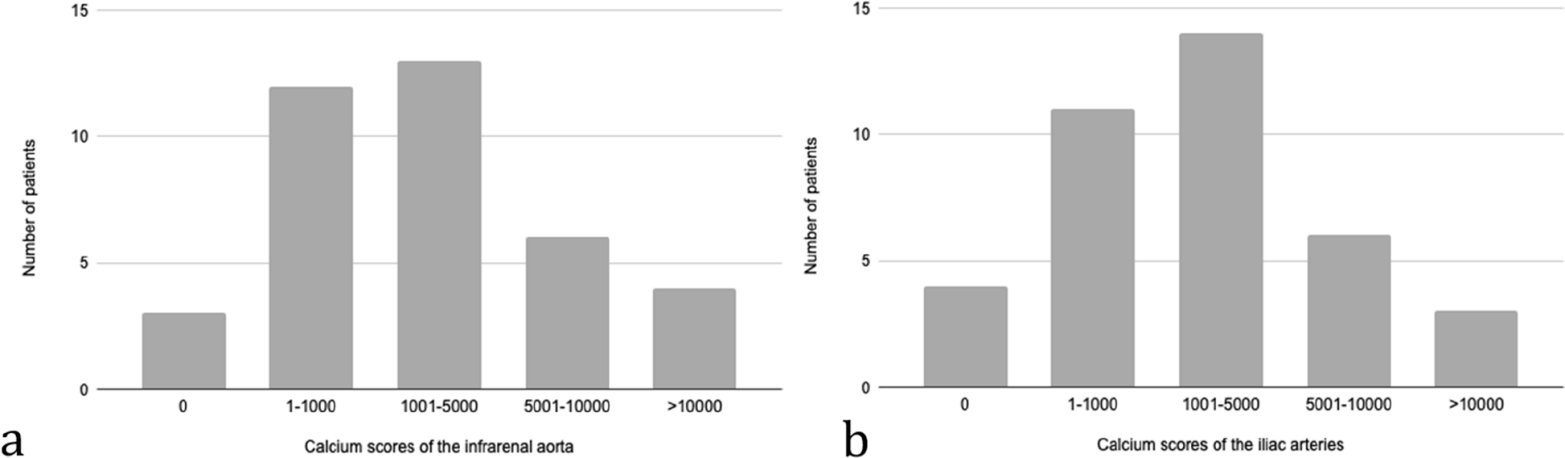
Histograms illustrating the distribution of the calcium scores measured on non-contrast CT scans of (3a) the infrarenal aorta and (3b) the iliac arteries

In total, scans of 38 kidney transplant candidates were analyzed; one non-contrast CT and one CT-angiography of each patient (Fig. 1). Mean age was 55 years, 66% were male, and 13% had established cardiovascular disease (Table 2). Non-contrast CT scans revealed vascular calcification of the infrarenal aorta in 92% and of the iliac arteries in 90% of the analyzed kidney transplant candidates. The median calcification scores were higher in the iliac arteries than the aorta (Table 2) with the majority of scores being in the lower range (Fig. 2).

**Table 2:** Baseline characteristics of the kidney transplant candidates

**Patient characteristic**	*n* = 38
Age (years)	55 ± 11.4
Sex, male	25 (66%)
Body mass index (kg/m^2^)	26 ± 4.5
Risk factors	
Dyslipidemia^a^	17 (45%)
Diabetes mellitus	10 (26%)
Hypertension	35 (92%)
Smoking	6 (16%)
Established cardiovascular disease^b^	5 (13%)
Dialysis for more than 1 year	15 (40%)
eGFR if preemptive (n = 22) (ml/min/1.73m^2^)	11 [9-12]
Dialysis status	
Hemodialysis	12 (32%)
Peritoneal dialysis	4 (11%)
Calcium score of the infrarenal aorta	
CT-angiography	2934 [771–11399]
Non-contrast CT scans	1671 [587–5053]
Calcium score of the iliac arteries	
CT-angiography	4540 [695–11739]
Non-contrast CT scans	1783 [186–4946]

Values are presented as mean ± SD, n (%) or median [IQR]

^a^Dyslipidemia was defined as total cholesterol > 6.2 mmol/l (240 mg/dl) or statin treatment. ^b^Established cardiovascular disease included previous myocardial infection (n = 1), stroke (n = 2), elective revascularization (n = 3), transitory cerebral ischemia, peripheral artery disease, or intermittent claudication

The CV% of the total calcium scores (infrarenal aorta + iliac arteries) was 22% when obtained from CT-angiographies and 14% when obtained from non-contrast CT scans, while the interobserver variabilities in CT-angiographies and non-contrast CT scans were 0.96 and 0.99, respectively (Table 3).

**Table 3:** The coefficient of variance (CV) and the interobserver variabilities

	CV%	Pearson correlation
Total calcium scores (infrarenal aorta + iliac arteries)		
CT-angiography	22	0.96
Non-contrast CT scans	14	0.99
The iliac arteries		
CT-angiography	38	
Non-contrast CT scan	33	
The infrarenal aorta		
CT-angiography	33	
Non-contrast CT scan	11	

**Fig. 3:**
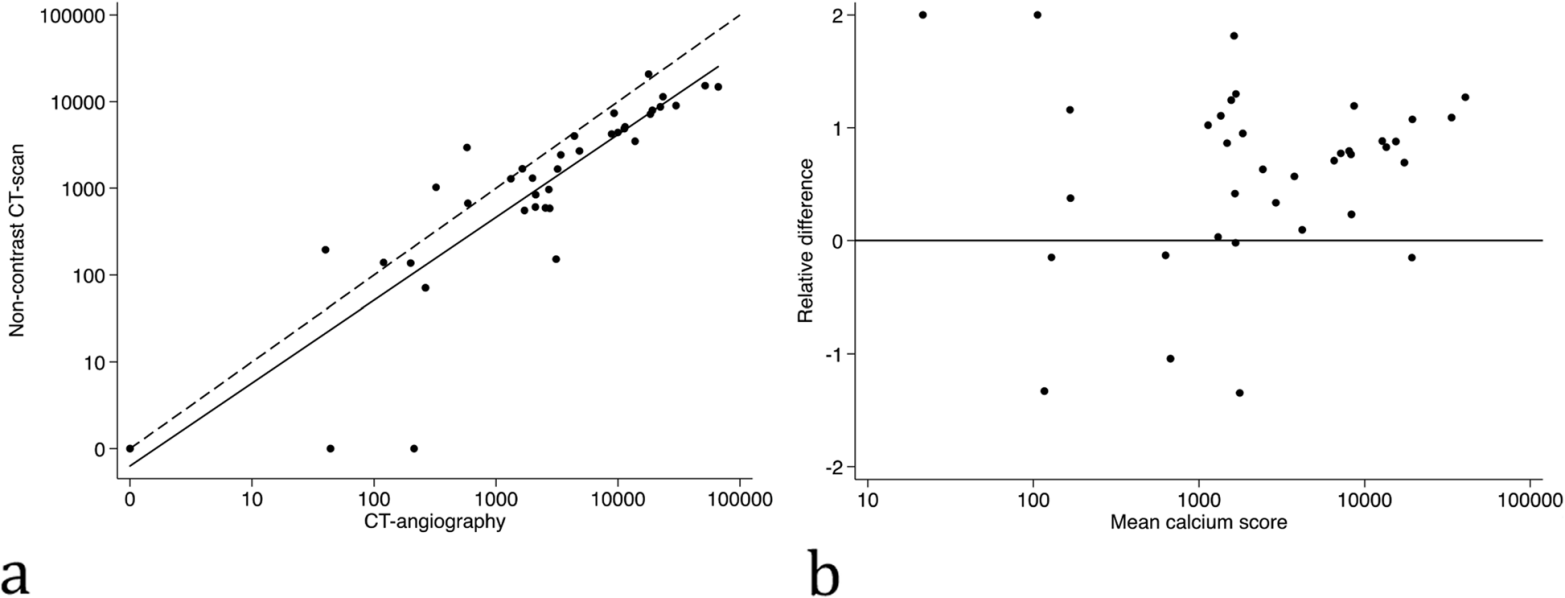
Calcium scores of the infrarenal aorta. **A**) XY-plot between calcium scores measured on a CT-angiography versus a non-contrast CT scan. Solid line = fitted regression line. Dashed line = equality line. **B**) Bland–Altman plot comparing mean calcium score to the relative difference between measurements on a CT-angiography and a non-contrast CT-scan

**Fig. 4:**
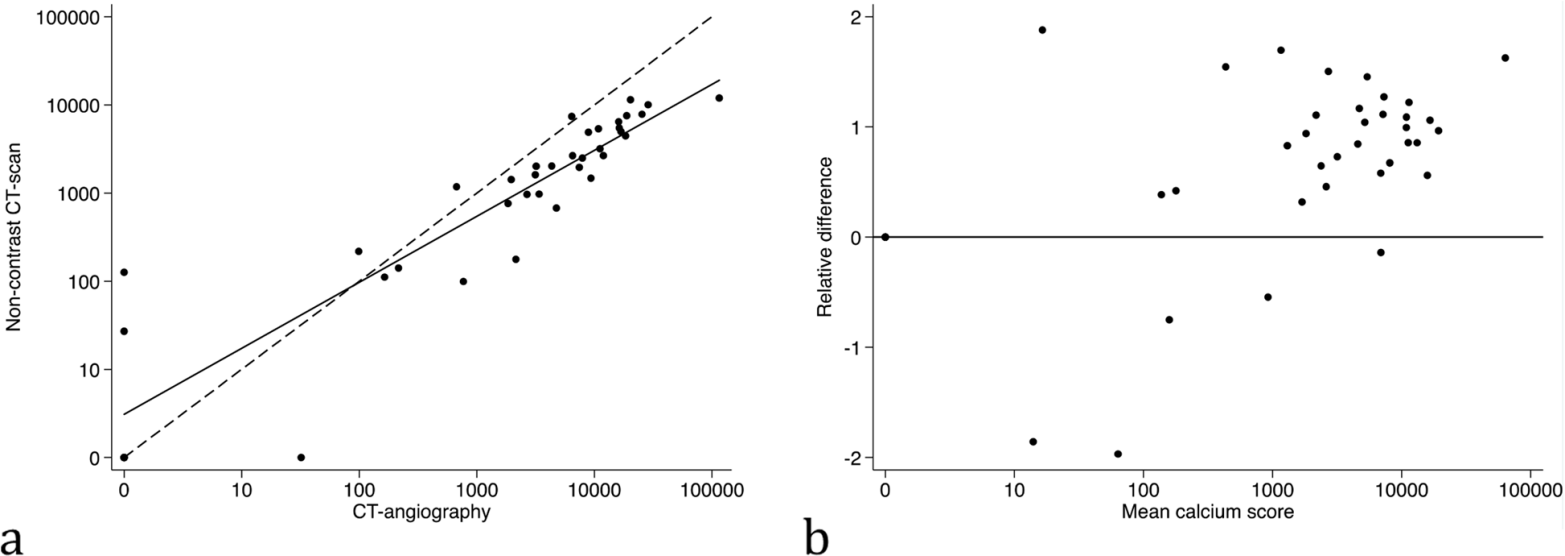
Calcium scores of the iliac arteries. A) XY-plot between calcium scores measured on a CT-angiography versus a non-contrast CT scan. Solid line = fitted regression line. Dashed line = equality line. B) Bland–Altman plot comparing mean calcium score to the relative difference of measurements on a CT-angiography and a non-contrast CT-scan

A linear relationship was observed between calcium scores from non-contrast CT scans and CT-angiographies both for the infrarenal aorta (R^2^ = 0.71, p < 0.0001) (Fig. 3a) and the iliac arteries (R^2^ = 0.71, p < 0.0001) (Fig. 4a). The calcium scores obtained from the CT-angiographies were generally higher than the calcium scores obtained from the non-contrast CT scans (Figs. 3b and 4b). The median differences were 1517 [48 – 6138] for the infrarenal aorta, and 2361 [59 – 8644] in the iliac arteries.

A correlation was observed between calcium volumes obtained from CT-angiography and non-contrast CT in both the infrarenal aorta (R^2^ = 0.67, p < 0.0001) and the iliac arteries (R^2^ = 0.76, p < 0.0001), but calcium volumes from the CT-angiographies were generally lower compared to non-contrast CT scans (Supplemental Fig. 1). The median differences were 946 mm^3^ [116 – 2807] for the infrarenal aorta, and 387 mm^3^ [56 – 1797] for the iliac arteries.

**Fig. 5:**
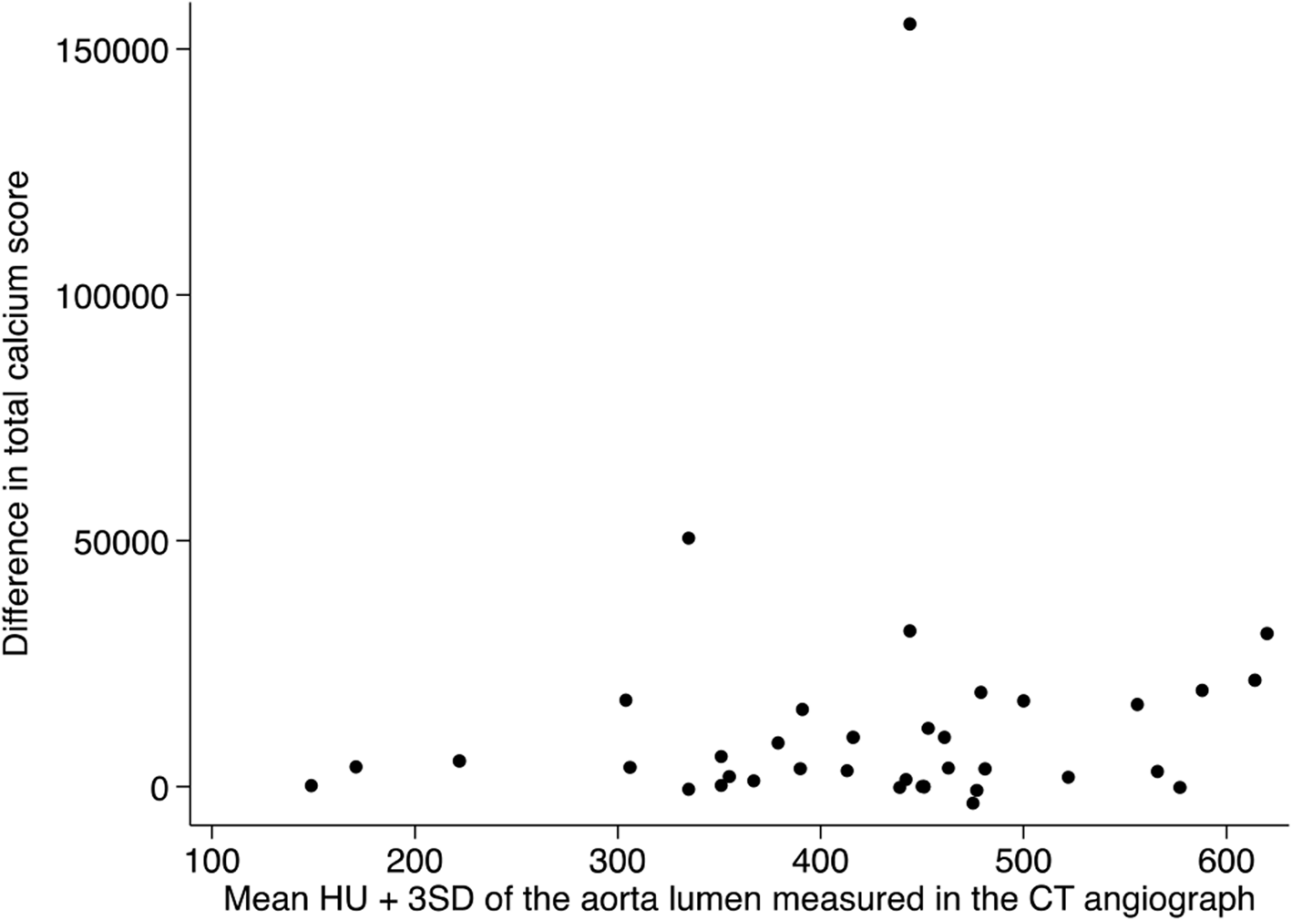
Differences in calcium scores between non-contrast CT and CT-angiography by the threshold (mean HU + 3 SD) obtained from the CT-angiography

There was no association between the difference in calcium scores between the two scans and the individual attenuation thresholds applied when analyzing CT-angiographies (Fig. 5).

## Discussion

In this cohort of kidney transplant candidates, calcium scores estimated from a contrast enhanced CT-angiography correlated well with calcium scores measured using non-contrast CT scans, albeit with higher absolute calcium scores. As part of the work-up prior to kidney transplantation, candidates may undergo multiple imaging [35] to examine vascular calcification and stenoses of the iliac arteries. The present findings suggest that using CT-angiography both stenoses and a calcium score may be estimated from the same scan allowing for additional risk assessment prior to kidney transplantation as aortoiliac calcium scores have been shown to predict cardiovascular events [11]. This may be included in the evaluation of eligibility for kidney transplantation.

The significant relationship observed between the calcium scores obtained from non-contrast CT scans and from CT-angiographies was consistent both for the infrarenal aorta and the iliac arteries when using individual attenuation thresholds for each patient based on the contrast density of the aortic lumen. The calcium scores obtained from the CT-angiographies were, however, higher than those obtained from the non-contrast CT scans. This is likely due to intramural contrast contamination causing higher densities and scores of the calcium containing areas on the CT-angiographies. This is despite the higher individual attenuation thresholds applied on the CT-angiographies compared to the standard threshold of non-contrast CT scans. In addition, it is plausible that the contrast that overlap the periphery of the calcium plaques may have increased the attenuation of calcium. It is also possible that some of the contrast-enhanced voxels that closely surround the calcium deposits might have been indistinguishable from and thus included in the calcium deposits on CT-angiographies. The lower estimated calcium volume from CT-angiographies, however, show that the total areas of presumed calcification are in fact lower when obtained from CT-angiographies compared to non-contrast CT scans. This is supported by the greater scattering of calcium volumes between the two scans at lower calcium volumes. Thus, the selection of the most appropriate attenuation threshold when analyzing the CT-angiographies must distinguish between calcium and contrast and may be further optimized in the future.

Quantitating calcium scores on CT-angiographies may add prognostic information in addition to the information obtained from assessing vascular stenoses [27, 38]. To further examine the possibility of implanting a kidney graft in the individual patient, information on the localization and local extent of calcifications is essential. This was, however, beyond the scope of the present paper, which aimed to assess if CT-angiography may be used to assess vascular calcification in the iliac vessels in general. Also, given the considerable variation some caution is warranted when applying calcium scores obtained from a CT-angiography, and future studies should aim to further standardize the methodology and examine the prognostic value of vascular calcification determined by CT-angiography.

The paired study design is a strength of this study as the patients underwent both non-contrast CT scan and CT-angiography on the same day. The findings are limited by the small cohort size. Also, as all included patients were kidney transplant candidates, extrapolation of the results to other cohorts should be done with caution. Importantly, we were not able to validate calcium scores obtained from the non-contrast CT and CT-angiographies with respect to the actual arterial wall calcium content. Additionally, the interobserver variability (in both non-contrast CT scans and CT-angiographies) is relatively large and may limit the reproducibility. As the study was performed as a post-hoc analysis, it was not possible to change the CT settings. The accuracy may have been improved using dual energy and a lower pitch.

In conclusion, the findings of this study suggest that CT-angiography images may be used to quantitate arterial calcification, which may improve risk stratification prior to kidney transplantation. The higher calcium score and large interobserver variability of the contrast-enhanced images when compared to non-contrast CT-scans suggest a need for further standardization, including the identification of the optimal attenuation thresholds for analysis of calcium scores from CT-angiographies.

## Supplementary Information

Additional file 1. Supplemental Fig. 1 The association between a) aortic and b) iliac artery calcium volumes (mm3) obtained from non-contrast CT and CT-angiography. Solid line = fitted regression line. Dashed line = equality line.

## Data Availability

All data generated and analyzed during this study are included or referred to in this published article.
