# Supplementary material for: Assessment of aortic and iliac artery calcification using CT-angiography in kidney transplant candidates
Source: CVIR Endovasc. 2025 May 6;8:39. doi: 10.1186/s42155-025-00542-1 (PMC12055728; doi:10.1186/s42155-025-00542-1)
Supplement: Supplementary file 1 — Additional file 1. Supplemental Fig. 1 The association between a) aortic and b) iliac artery calcium volumes (mm3) obtained from non-contrast CT and CT-angiography. Solid line = fitted regression line. Dashed line = equality line. [file 42155_2025_542_MOESM1_ESM.docx]

**Supplementary material****

Fig. 1a Fig. 1b

**Supplemental Fig. 1** The association between a) aortic and b) iliac artery calcium volumes (mm^3^) obtained from non-contrast CT and CT-angiography. Solid line = fitted regression line. Dashed line = equality line.
